# Pharmacologic attenuation of pelvic pain in a murine model of interstitial cystitis

**DOI:** 10.1186/1471-2490-9-16

**Published:** 2009-11-12

**Authors:** Charles N Rudick, Anthony J Schaeffer, David J Klumpp

**Affiliations:** 1 Department of Urology, Feinberg School of Medicine, Northwestern University, Chicago, Illinois, USA; 2 Department of Microbiology-Immunology, Feinberg School of Medicine, Northwestern University, Chicago, Illinois, USA

## Abstract

**Background:**

Interstitial cystitis/painful bladder syndrome (IC/PBS) is a bladder disease that causes debilitating pelvic pain of unknown origin, and IC/PBS symptoms correlate with elevated bladder lamina propria mast cell counts. Similar to IC/PBS patients, pseudorabies virus (PRV) infection in mice induces a neurogenic cystitis associated with bladder lamina propria mast cell accumulation and pelvic pain. We evaluated several drugs to determine the effectiveness of reducing PRV-induced pelvic pain.

**Methods:**

Neurogenic cystitis was induced by the injection of Bartha's strain of PRV into the abductor caudalis dorsalis tail base muscle of female C57BL/6 mice. Therapeutic modulation of pelvic pain was assessed daily for five days using von Frey filament stimulation to the pelvic region to quantify tactile allodynia.

**Results:**

Significant reduction of PRV-induced pelvic pain was observed for animals treated with antagonists of neurokinin receptor 1 (NK1R) and histamine receptors. In contrast, the H1R antagonist hydroxyzine, proton pump inhibitors, a histamine receptor 3 agonist, and gabapentin had little or no effect on PRV-induced pelvic pain.

**Conclusion:**

These data demonstrate that bladder-associated pelvic pain is attenuated by antagonists of NK1R and H2R. Therefore, NK1R and H2Rrepresent direct therapeutic targets for pain in IC/PBS and potentially other chronic pain conditions.

## Background

IC/PBS is a chronic bladder inflammatory disease with unknown etiology that afflicts as many as 1 million patients in the United States and is associated with severe pelvic pain and voiding dysfunction that includes urinary frequency, urgency, and nocturia [1-3]. Clinical studies demonstrate elevated mast cell numbers in the lamina propria of IC/PBS bladder biopsies, and the partial efficacy of neuromodulatory therapies suggests neural-immune interactions play a role in IC/PBS associated pelvic pain ([4,5] and reviewed in [1]). Mast cells contain preformed stores of immune mediators, such as histamine, and can be activated to release these stores by neurotransmitters such as substance P. These observations have suggested a central role for mast cells in IC/PBS pathogenesis whereby activation of bladder-associated circuits in the central nervous system initiates substance P release by peripheral nerves in the bladder that then promotes substance P-mediated mast cell activation [6]. This mast cell activation, in turn, could release histamine that acts on nociceptive neurons to induce pelvic pain that originates from the bladder.

In support of this hypothesis, a subset of IC/PBS patients exhibit increased density of substance P fibers in the bladder sub mucosa with substance P fibers in close proximity to mast cells [7]. Furthermore, accumulation of lamina propria mast cells is correlated with IC/PBS symptoms, and increased levels of urinary histamine metabolites have been detected in IC/PBS patient urines [4,5,8,9]. These studies suggest that antihistamine treatment may alleviate IC/PBS associated symptoms, however clinical trials report varying results. Pilot clinical studies yielded modest pain relief in IC/PBS patients receiving the old-line H1R antagonist hydroxyzine [10], but studies have not yet been conducted with newer generation H1R antagonists. In contrast, the H2R antagonist cimetidine produced significant improvement in pain and nocturia in a limited trial of PBS patients [11]. In agreement with these clinical findings, a murine interstitial cystitis model has demonstrated a requirement for mast cells and histamine receptors 1 and 2 in pelvic pain originating from the bladder (Table 1) [12,13].

**Table 1:** Comparison of murine neurogenic cystitis with human IC/PBS.

	IC/PBS	Murine Cystitis	References
**Pelvic Specific Pain**	Yes	Yes	[1,12,13]
**Role for mast cells**	Yes	Yes	[4,5,12,16,18,19]
**Role for substance P**	Yes	Yes	[7], This paper
**Role for histamine**	Yes	Yes	[9,12]

Jasmin and colleagues were the first to show that the attenuated Bartha's strain of pseudorabies virus (PRV) causes cystitis in rats when injected into the tailbase *abductor caudalis dorsalis *muscle [14,15]. PRV is an α-herpesvirus that is taken up by motor neurons and undergoes retrograde transport to the central nervous system (CNS) where the virus replicates. PRV-induced cystitis is a neurally mediated cystitis even though Bartha's PRV is incapable of descending the sensory nerves to the bladder [13-17]. In mice, PRV causes cystitis in the form of bladder-specific pathophysiology which includes focal inflammation, lamina propria mast cell accumulation, the formation of apoptotic lesions in the urothelium, and a marked loss of urothelial barrier function [18,19]. In addition, murine PRV-induced cystitis was also shown to induce pain specific to the pelvic region in female mice [13]. This pelvic pain behavior was reduced by intravesical lidocaine but not by intrauterine lidocaine, suggesting that pelvic pain behavior was associated with the bladder. Interestingly, colonic lidocaine also relieved pain despite an absence of detectable bowel pathology, supporting previous observations of physiologic crosstalk between bladder and bowel and consistent with food sensitivities exhibited by IC/PBS patients.

Here, we examined the therapeutic effects of multiple drugs on pelvic pain in an established murine model that recapitulates key aspects of IC/PBS, including lamina propria mast cell accumulation and pelvic pain (see Table 1) [13,16,18,19]. Pharmacologic antagonism of H2R, H1R or NK1R attenuated pelvic pain, demonstrating that PRV-induced pelvic pain is modulated through blocking the actions of histamine or substance P. In contrast, the H1R antagonist hydroxyzine, proton pump inhibitors, a histamine receptor 3 agonist, and gabapentin had little or no effect on PRV-induced pelvic pain. Thus, new generation H1 antihistamines, H2 antihistamines and NK1R antagonists are candidates for expanded clinical trials in the treatment of IC/PBS associated pain.

## Methods

### Animals

Adult female C57BL/6J mice (10-14 weeks old) were purchased from Jackson Laboratory (Bar Harbor, ME). All experiments were performed using protocols approved by Northwestern University Animal Care and Use Committee. Mice were housed in containment facilities of the Center for Comparative Medicine and maintained on a regular 12:12 hour light:dark cycle with food and water *ad libitum*.

### Induction of neurogenic cystitis

Pseudorabies virus (PRV) was prepared and titrated as previously reported in Chen et al 2006 [18]. Neurogenic cystitis was induced by injection of 2.29 × 10^6 ^pfu Bartha's PRV through the skin into the abductor caudalis dorsalis (ACD) muscle using a 26 gauge Hamilton syringe while maintaining the animals under Isoflurane anesthesia.

### Behavioral Testing

Mice were tested prior to PRV administration (baseline), 1, 2, 3 and 4 post infection days (PID) after PRV inoculation. Referred hyperalgesia and tactile allodynia was tested using von Frey filaments applied to the abdomen [20]. Mice were tested in individual Plexiglas chambers (6 cm × 10 cm × 12 cm) with a stainless steal wire grid floor (mouse acclimation period of ~10 min prior to testing). Frequency of withdrawal responses to the application of von Frey filaments to the abdomen was tested using five individual fibers with forces of 0.04, 0.16, 0.4, 1 and 4 grams (Stoelting, USA). Each filament was applied for ~1 second with an inter-stimulus interval of 2-5 s for a total 10 times, and the hairs were tested in ascending order of force. Stimulation was confined to the lower abdominal area in the general vicinity of the bladder and care was taken to stimulate different areas within this region to avoid desensitization or "wind up" effects. Three types of behaviors were considered as positive responses to filament stimulation: (1) sharp retraction of the abdomen; (2) immediate licking or scratching of the area of filament stimulation; or (3) jumping.

### Therapeutic Treatment

Therapy was initiated 1 hour prior to PRV inoculation and was repeated every 24 hours until PID 4. Drugs were administered using the following dosages that were previously demonstrated as efficacious in murine models: 10 mg/kg Diphenhydramine, 10 mg/kg hydroxyzine, 10 mg/kg cetirizine, 10 mg/kg cimetidine, 10 mg/kg ranitidine, 10 mg/kg famotidine, 50 mg/kg gabapentin, 12 mg/kg L-703,606, 5 mg/kg lansoprazole, 5 mg/kg omeprazole and 10 mg/kg imetit (Sigma, St. Louis, MO) was administered by either oral gavage (Hamiltion syringe with a rounded tip needle 2.5 cm long) or I.P. (gabapentin, imetit and L-703,606) [21-25]. PRV-infected mice and sham controls were gavaged with saline until PID4. All mice were tested for referred hyperalgesia using von Frey filaments before PRV (baseline) and PID1-4.

### Statistical analyses

Results were expressed as mean ± SEM and analyzed for statistical significance by single factor ANOVA compared to baseline values for each filament per group. A value of p < 0.05 was considered statistically significant.

## Results

### H1R antihistamine effects on PRV-induced pelvic pain

**Figure 1:**
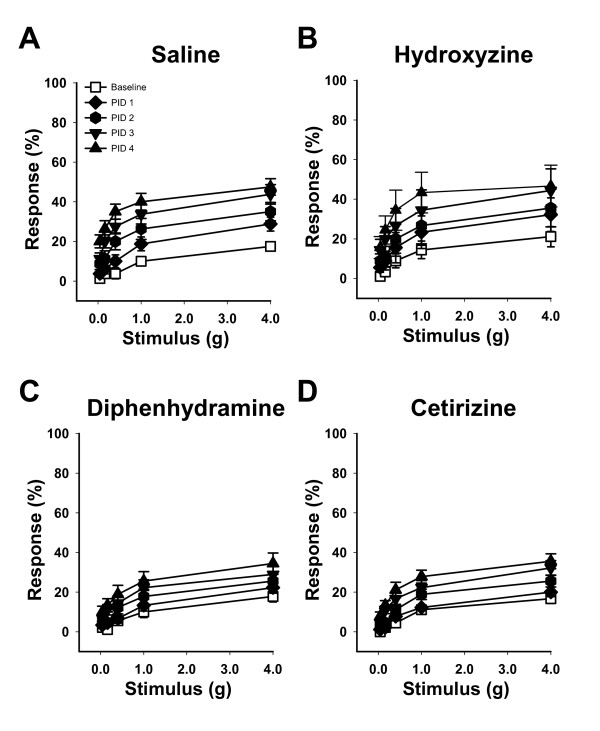
**Antihistamines specific for H1 receptor attenuate pelvic pain**. **A) **Female mice treated by oral gavage with saline exhibited significantly increased pelvic sensitivity relative to baseline at PID 2, 3 and 4 for all filaments tested (n = 8, P < 0.05). **B) **Female mice treated by oral gavage with the H1R antagonist hydroxyzine showed a modest reduction in pelvic sensitivity that was significantly increased above baseline by PID 2, 3 and 4 (n = 9, P < 0.05). **C) **Female mice treated by oral gavage with the H1R antagonist diphenhydramine showed reduced pelvic sensitivity that was not significantly increased above baseline until PID 3 and 4 (n = 9, P < 0.05). **D) **Female mice treated by oral gavage with the H1R antagonist cetirizine showed reduced pelvic sensitivity that was not significantly increased above baseline until PID 3 and 4 (n = 9, P < 0.05). The symbol key shown in (A) applies to panels A-D. Error bars depict SEM, and significance was determined by ANOVA.

Pain originating from a visceral organ is typically referred to a corresponding "dermatome" on the skin that shares common spinal cord innervation with the given visceral organ [26]. A role for histamine and histamine receptors in pain responses has been documented in both humans and animal models [10,12,27]. To examine H1R as a therapeutic target for pelvic pain, we examined the effects of pharmacologic antagonists on the development of PRV-induced pain responses. PRV-infected C57BL/6 female mice were treated by oral gavage with saline or with antihistamines specific for H1R (hydroxyzine, diphenhydramine, or cetirizine). Mice treated with saline alone exhibited responses to pelvic stimuli that were significantly greater than baseline by PID 2, 3 and 4 (Figure 1A; P < 0.05). Mice treated with the H1R antagonist hydroxyzine showed a modest reduction in pelvic sensitivity (Table 2) that was significantly increased above baseline by PID 2, 3 and 4 (Figure 1B; P < 0.05). In contrast, diphenhydramine-treated mice failed to develop significant pain until PID 3 and 4 (Figure 1C; P < 0.05) and developed only 56% of the total pain exhibited by saline-treated mice (Table 2). Similar to diphenhydramine-treated mice, cetirizine-treated mice failed to develop significant pain until PID 3 and 4 (Fig 1D; P < 0.05) and developed only 59% of the total pain exhibited by saline-treated mice (Table 2).

**Table 2:** Therapeutic modulation of PRV-associated pelvic pain.

Group	% Increase*	% Relief**	P-Value**
**Experiment 1**			
**Saline**	392.4 ± 39.6	0	
**Hydroxyzine**	383.9 ± 123.9	2	P < 0.96
**Diphenhydramine**	222.4 ± 74.7	44	P < 0.08
**Cetirizine**	230.6 ± 52.8	41	P < 0.06
**Experiment 2**			
**Saline**	431.7 ± 92.4	0	
**Cimetidine**	182.4 ± 43.3	58	**P < 0.02**
**Ranitidine**	101.0 ± 37.5	77	**P < 0.005**
**Famotidine**	167.3 ± 35.1	61	**P < 0.02**
**Experiment 3**			
**Saline**	498.8 ± 112.7	0	
**Gabapentin**	371.7 ± 136.7	25	P < 0.50
**L-703,606**	116.7 ± 55.8	77	**P < 0.02**
**Experiment 4**			
**Saline**	388.1 ± 69.5	0	
**Lansoprazole**	394.0 ± 44.7	0	P < 0.95
**Omeprazole**	368.7 ± 68.6	5	P < 0.85
**Imetit**	454.3 ± 111.8	0	P < 0.63

*at PID4 relative to baseline **at PID4 relative to saline

### Antihistamines specific for H2R significantly attenuate PRV-induced pelvic pain

**Figure 2:**
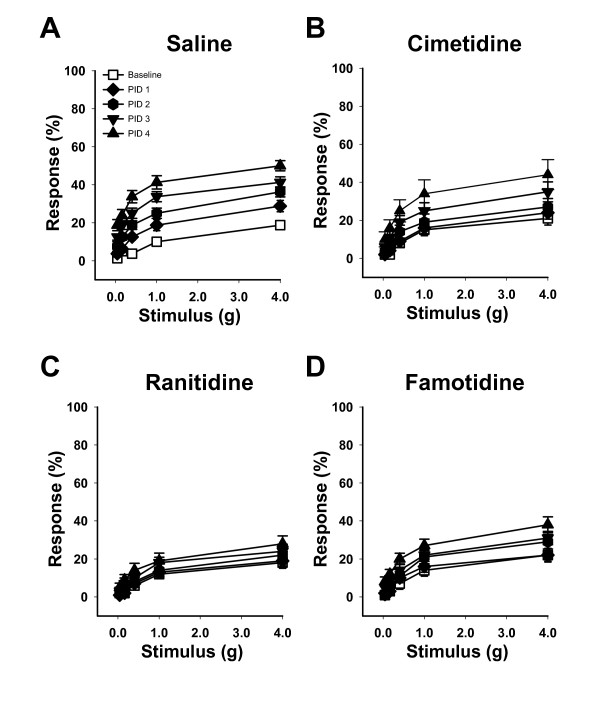
**Antihistamines specific for H2 receptor attenuate pelvic pain**. **A) **Female mice treated by oral gavage with saline exhibited significantly increased pelvic sensitivity relative to baseline at PID 2, 3 and 4 for all filaments tested (n = 8, P < 0.05). **B) **Female mice treated by oral gavage with the H2R antagonist cimetidine showed reduced pelvic sensitivity that was not significantly increased above baseline until PID 4 (n = 10, P < 0.05). **C) **Female mice treated by oral gavage with H2R antagonist ranitidine did not develop significant pelvic sensitivity (n = 10). **D) **Female mice treated by oral gavage with the H2R antagonist famotidine showed reduced pelvic sensitivity that was not significantly increased above baseline until PID 4 (n = 10, P < 0.05). The symbol key shown in (A) applies to panels A-D. Error bars depict SEM, and significance was determined by ANOVA.

To evaluate histamine 2 receptor as a therapeutic target for pelvic pain, we examined the effects of pharmacologic antagonists on the development of PRV-induced pain responses. PRV-infected C57BL/6 female mice were treated by oral gavage with saline or with antihistamines specific for H2R (cimetidine, ranitidine or famotidine). Mice treated with saline alone exhibited responses to pelvic stimuli that were significantly greater than baseline by PID 2, 3 & 4 (Figure 2A; P < 0.05). Cimetidine-treated mice failed to develop significant pain until PID 4 (Figure 2B; P < 0.05) and developed only 42% of the pain exhibited by saline-treated mice (Table 2). In contrast, ranitidine-treated mice showed no significant pain responses relative to baseline at any timepoint (Figure 2C; P > 0.1) and developed only 23% of the pain exhibited by saline-treated mice (Table 2). Similar to Cemetidine-treated mice, famotidine-treated mice failed to develop significant pain until PID 4 (Figure 2D; P < 0.05) and developed only 49% of the pain exhibited by saline-treated mice (Table 2). These data demonstrate that PRV-induced pelvic pain can be significantly attenuated by H2R antagonists.

### Neural therapies differentially attenuate PRV-induced pelvic pain

**Figure 3:**
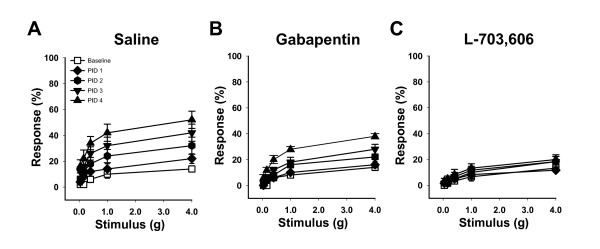
**Substance P receptor antagonist and gabapentin attenuate pelvic pain**. **A) **Female mice treated with saline (I.P.) exhibited significantly increased pelvic sensitivity relative to baseline at PID 2, 3 and 4 for all filaments tested (n = 5, P < 0.05). **B) **Female mice treated with gabapentin (I.P.) showed reduced pelvic sensitivity that was not significantly increased above baseline until PID 3 (n = 5, P < 0.05). **C) **Female mice treated with a substance P receptor antagonist L-703,606 did not develop significant pelvic sensitivity (n = 6). The symbol key shown in (A) applies to panels A-C. Error bars depict SEM, and significance was determined by ANOVA.

We examined an NK1R antagonist and gabapentin on the development of PRV-induced pain responses. PRV-infected C57BL/6 female mice were treated I.P. with saline, the NK1R antagonist L-703,606 or gabapentin. Mice treated with saline alone exhibited responses to pelvic stimuli that were significantly greater than baseline by PID 2, 3 & 4 (Figure 3A; P < 0.05). Gabapentin-treated mice failed to develop significant pain until PID 3 and 4 (Fig 3B; P < 0.05) and developed 74% of the pain exhibited by saline-treated mice (Table 2). In contrast, L-703,606-treated mice showed no significant pain responses relative to baseline at any timepoint (Figure 3C; P > 0.1) and developed only 23% of the pain exhibited by saline-treated mice (Table 2), similar to ranitidine-treated mice. These data demonstrate that PRV-induced pelvic pain can be significantly attenuated by a NK1R antagonist.

### Proton pump inhibitors have minimal or no effect on PRV-induced pelvic pain

To evaluate proton pumps and H3R agoninsts as therapeutic targets for pelvic pain, we examined the effects of pharmacologic antagonists on the development of PRV-induced pain responses. PRV-infected C57BL/6 female mice were treated by oral gavage with saline, with proton-pump inhibitors (lansoprazole or omeprazole) or an H3R agonist (imetit). Mice treated with saline alone exhibited responses to pelvic stimuli that were significantly greater than baseline by PID 2, 3 & 4 (data not shown). Mice treated with the lansoprazole, omeprazole or imetit showed little or no reduction in pelvic sensitivity (Table 2) that was significantly increased above baseline by PID 3 (data not shown). These data demonstrate that PRV-induced pelvic pain is not significantly attenuated by proton-pump inhibitors or H3R agonist.

## Discussion

In a murine model of IC/PBS that recapitulates pelvic specific pain, we find that pelvic pain can be modulated by the neural-immune axis through blocking the actions of histamine or substance P. Previously, we showed that PRV-induced pelvic pain is mediated by mast cells via H1R and H2R, using both genetic and pharmacologic approaches [12]. We expanded these observations in this study by demonstrating that both famotidine and cimetidine can attenuate pelvic pain similar to ranitidine, however ranitidine is the most effective, reducing 77% of the pelvic pain (Table 2). We also find that the substance P receptor antagonist, L-703,606, attenuated pelvic pain by 77% demonstrating it to be as effective a ranitidine (Table 2). Furthermore blocking H1R with diphenhydramine and cetirizine reduced pelvic pain by at least 40%, whereas the old generation H1R antagonist hydroxyzine had only a modest reduction in pelvic pain (Table 2). In contrast to our previous study, however, diphenydramine pain blockade failed to achieve statistical robustness here (P < 0.08) due to unusual variability. One possible influence is unforeseen environmental factors, as the animal containment facilities necessary for PRV studies have relocated to a new building. Nonetheless, while these results emphasize the statistical vagaries inherent in small animal sample sizes, the findings here are consistent with our previous report. Conversely, proton pump inhibitors, histamine receptor 3 agonist, and gabapentin had little or no effect on PRV-induced pelvic pain (Table 2). These data identify histamine 1 and 2 receptors and neurokinin 1 receptor as therapeutic targets for direct intervention in pelvic pain of IC/PBS.

Our data support a model of IC/PBS pathogenesis involving a positive feedback loop as originally postulated by Theoharides and Elbadawi, whereby substance P-containing peripheral nerves stimulate mast cells, in turn releasing histamine that induces pain originating from the bladder [6,9]. Furthermore, histamine release by mast cells feeds back onto peripheral nerves to cause sustained release of substance P in the periphery to prolong mast cell activation or in the spinal cord to prolong neuronal excitation. This model is consistent with murine neurogenic cystitis and a subset of IC/PBS patients [7]. We find that the substance P receptor antagonist, L-703,606, attenuated pelvic pain behavior by 77% (Table 2). L-703,606, could be acting in the periphery to block substance P induction of mast cell-derived histamine and subsequent activation of pain fibers. Alternatively, L-703,606 could be acting in the spinal cord to block neuron-derived substance P activation of dorsal horn neurons that mediate pain. Regardless of the mechanism, our data demonstrate that blocking substance P attenuates pelvic pain in murine neurogenic cystitis and is a novel therapeutic target for direct intervention in pelvic pain.

We previously observed isolectin B4-positive processes within the lamina propria, where mast cells accumulate in IC/PBS and our murine IC model [4,5,18], suggesting that interactions between mast cell histamine and C fiber nociceptors mediate pelvic pain in this IC/PBS model. This is reminiscent of an esophagitis model where mast cell histamine stimulates C fiber activity via H1R, therefore it is possible that histamine may act on bladder sensory nerves in IC/PBS because histamine metabolites are increased in IC/PBS urine [9,28]. This mechanism is similar to patients with irritable bowel syndrome where abdominal/pelvic pain is correlated with activated colonic mucosal mast cells in proximity to nerves, and colonic tissue extracts that contained elevated histamine that excited rat nociceptors [29]. These findings support the idea that neural-immune interactions can mediate pain in multiple pelvic pain syndromes.

Our evidence for histamine-mediated pelvic pain provides a mechanistic understanding of previous clinical findings. The H2R antagonist cimetidine produced significant improvement in pain and nocturia in a limited trial of IC/PBS patients [11], however clinical trials have yet to be conducted with ranitidine or famotidine. Similarly, pilot clinical studies yielded modest pain relief in IC/PBS patients receiving the old-line H1R antagonist hydroxyzine (Atarax) [10], but studies have not yet been conducted with newer generation H1R antagonists like diphenhydramine or cetirizine. Modern H1R antagonists likely attenuated pelvic pain better here than older generation H1R antagonists because newer H1R antagonists are well-absorbed, act faster, have greater efficacy, and a longer duration of action [30]. We have previously shown that H1R and H2R are expressed in both the bladder and colon of mice, leaving the possibility of the H1R and H2R antihistamines acting at either or both of the organs to quell pelvic pain. Additionally, the colon modulates PRV-induced pelvic pain, pelvic organs generally exhibit neural-mediated crosstalk, and IBS is a co-morbidity of IC/PBS [13,31]. Therefore, these epidemiologic and animal model findings together suggest that H1R and H2R antagonists may have general application for the treatment of pelvic pain.

## Conclusion

These findings support a general model for IC/PBS pelvic pain due to mast cell-sensory nerve interactions. Pharmacologic antagonism of H1R, H2R or NK1R attenuated pelvic pain, blocking the actions of histamine or substance P represent therapeutic targets for direct intervention in pelvic pain. Thus, new generation H1 antihistamines, H2 antihistamines and NK1R antagonists are candidates for expanded clinical trials in the treatment of pain associated with IC/PBS.

## Competing interests

The authors declare that they have no competing interests.

## Authors' contributions

CR participated in the design of the study, experimental testing and performed the statistical analysis. DK participated in the design of the study and statistical analysis. AS participated in the design of the study. All authors participated in manuscript preparation, read and approved the final manuscript.

## Pre-publication history

The pre-publication history for this paper can be accessed here:

http://www.biomedcentral.com/1471-2490/9/16/prepub
